# Genetic Profiling Using Genome-Wide Significant Coronary Artery Disease Risk Variants Does Not Improve the Prediction of Subclinical Atherosclerosis: The Cardiovascular Risk in Young Finns Study, the Bogalusa Heart Study and the Health 2000 Survey – A Meta-Analysis of Three Independent Studies

**DOI:** 10.1371/journal.pone.0028931

**Published:** 2012-01-25

**Authors:** Jussi A. Hernesniemi, Ilkka Seppälä, Leo-Pekka Lyytikäinen, Nina Mononen, Niku Oksala, Nina Hutri-Kähönen, Markus Juonala, Leena Taittonen, Erin N. Smith, Nicholas J. Schork, Wei Chen, Sathanur R. Srinivasan, Gerald S. Berenson, Sarah S. Murray, Tomi Laitinen, Antti Jula, Johannes Kettunen, Samuli Ripatti, Reijo Laaksonen, Jorma Viikari, Mika Kähönen, Olli T. Raitakari, Terho Lehtimäki

**Affiliations:** 1 Department of Clinical Chemistry, University of Tampere, Medical School, and Tampere University Hospital, Tampere, Finland; 2 Department of Clinical Physiology, University of Tampere, Medical School, and Tampere University Hospital, Tampere, Finland; 3 Department of Pediatrics, University of Tampere, Medical School, and Tampere University Hospital, Tampere, Finland; 4 Department of Surgery, University of Tampere, Medical School, and Tampere University Hospital, Tampere, Finland; 5 FIMM, Institute for Molecular Medicine Finland, Helsinki, Finland; 6 Department of Medicine, University of Turku and Turku University Hospital, Turku, Finland; 7 Department of Clinical Physiology, University of Turku and Turku University Hospital, Turku, Finland; 8 Research Centre of Applied and Preventive Cardiovascular Medicine, University of Turku and Turku University Hospital, Turku, Finland; 9 Department of Pediatrics, University of Oulu and Oulu University Hospital, Oulu, Finland and Department of Pediatrics, Vaasa Central Hospital, Vaasa, Finland; 10 Department of Clinical Physiology and Nuclear Medicine, University of Eastern Finland, Kuopio University Hospital, Kuopio, Finland; 11 Department of Health and Functional Capacity, National Public Health Institute, Turku, Finland; 12 The Scripps Translational Science Institute and Scripps Health, La Jolla, California, United States of America; 13 The Scripps Research Institute, La Jolla, California, United States of America; 14 Department of Epidemiology, Tulane University, New Orleans, Louisiana, United States of America; Heart Center Munich, Germany

## Abstract

**Background:**

Genome-wide association studies (GWASs) have identified a large number of variants (SNPs) associating with an increased risk of coronary artery disease (CAD). Recently, the CARDIoGRAM consortium published a GWAS based on the largest study population so far. They successfully replicated twelve already known associations and discovered thirteen new SNPs associating with CAD. We examined whether the genetic profiling of these variants improves prediction of subclinical atherosclerosis – i.e., carotid intima-media thickness (CIMT) and carotid artery elasticity (CAE) – beyond classical risk factors.

**Subjects and Methods:**

We genotyped 24 variants found in a population of European ancestry and measured CIMT and CAE in 2001 and 2007 from 2,081, and 2,015 subjects (aged 30–45 years in 2007) respectively, participating in the Cardiovascular Risk in Young Finns Study (YFS). The Bogalusa Heart Study (BHS; n = 1179) was used as a replication cohort (mean age of 37.5). For additional replication, a sub-sample of 5 SNPs was genotyped for 1,291 individuals aged 46–76 years participating in the Health 2000 population survey. We tested the impact of genetic risk score (GRS_24SNP/CAD_) calculated as a weighted (by allelic odds ratios for CAD) sum of CAD risk alleles from the studied 24 variants on CIMT, CAE, the incidence of carotid atherosclerosis and the progression of CIMT and CAE during a 6-year follow-up.

**Results:**

CIMT or CAE did not significantly associate with GRS_24SNP/CAD_ before or after adjusting for classical CAD risk factors (p>0.05 for all) in YFS or in the BHS. CIMT and CAE associated with only one SNP each in the YFS. The findings were not replicated in the replication cohorts. In the meta-analysis CIMT or CAE did not associate with any of the SNPs.

**Conclusion:**

Genetic profiling, by using known CAD risk variants, should not improve risk stratification for subclinical atherosclerosis beyond conventional risk factors among healthy young adults.

## Introduction

Current risk assessment strategies do not fully explain the factors underlying cardiovascular risk [1]. Emphasizing this, risk scores based on serum lipid levels and traditional factors such as smoking, hypertension, sex, etc., lack accuracy in risk prediction and have a tendency to overestimate the risk in low-risk populations and to underestimate the risk in high-risk populations [2]. The role of the hereditary component in the development of atherosclerosis should be studied further as the progression of the disease is also influenced by genetics. This could lead to improved risk assessment strategies.

The recent surge in genetic profiling with the aid of genome-wide association studies (GWASs) has found novel variants associating with myocardial infarction (MI) and coronary artery disease (CAD) [3], [4], [5], [6], [7], [8], [9], [10]. These variants could be biomarkers themselves or point to circulating markers for further exploration [11]. The most recent GWAS-based meta-analysis (the CARDIoGRAM consortium), with the largest population sample to date (∼22,000 cases and ∼65,000 controls with a replication sample of ∼57,000 individuals), revealed 13 new genome-wide -significant candidate loci associating with the occurrence of CAD and successfully replicated twelve previously discovered associations [3], [4], [5], [6], [7], [8], [9], [10], [12]. Although these CAD variants strongly associate with the risk of CAD, their potential role in the development of atherosclerosis is still obscure.

Atherosclerosis begins with a subclinical phase starting in childhood and young adulthood [13], [14]. Although it is the first stage of the disease, mainly manifested by an early intimal thickening and decreased elasticity of the arteries, it may be used to predict the future outcome of the disease. Increased subclinical atherosclerosis, as assessed by carotid artery intima-media thickness (CIMT) or elasticity (CAE), is a strong predictor of future cardiovascular events such as MI and stroke [2], [5], [8]. For this reason, the identification of individuals at high hereditary subclinical risk could be utilized in primary prevention [15], [16], [17]. Furthermore, it would be very useful to clarify the mechanisms by which these variants exert their effects on the risk of CAD. At present, only five SNPs associated with CAD in previous GWASs(rs10757274, rs1333049, rs6922269 rs501120 and rs2943634) have been investigated for possible associations with subclinical atherosclerosis [18], [19], [20], [21], [22]. Of these five, three variants are in complete or high linkage disequilibrium with two of the risk variants for CAD identified by the CARDIoGRAM consortium.

Given the current gap in knowledge, we tested the hypothesis that genetic profiling by using these novel CAD variants could be useful in the risk stratification for subclinical atherosclerosis (depicted by CAE and CIMT) before and after adjusting for traditional risk factors. Genetic information on these variants was used to form a genetic risk score depicting individual risk for CAD (GRS_SNP/CAD_). The possible effects of individual SNPs were also screened. For these analyses we used three representative population-based studies, i.e., The Cardiovascular Risk in Young Finns and The Bogalusa Heart Study. A subsample of the SNPs was also previously genotyped from the Health 2000 Survey cohort and the results were used for further replication.

## Results

### General characteristics of the study populations

In 2007, the mean age of the YFS population was 37.5 years (S.D. 5.0). There were 2,015 subjects with both ultrasound and genotyping data available. One of the subjects had established cardiovascular disease, 43 were on antihypertensive medication, and 7 (0.3%) were on lipid-lowering medication. The use of lipid medication was more frequent in the replication cohorts: 68 (5.8%) in the Bogalusa Heart Study and 128 (9.2%) in the Health 2000 survey. The general characteristics of the YFS population and replication cohorts are presented in Table 1. All of the 24 genotypes followed the Hardy-Weinberg equilibrium (p>0.05 for χ^2^ -test).

**Table 1:** General characteristics of the three study populations with available ultrasound and genotype data.

	Young Finns Study		Health 2000 Survey		The Bogalusa Heart Study	
	Men	Women	Men	Women	Men	Women
	n = 909	n = 1106	n = 625	n = 761	n = 499	n = 681
Age, years	37.5 (5.0)	37.5 (5.0)	58.0 (7.9)	58.6 (8.3)	37.8 (4.6)	37.3 (4.7)
BMI, kg/m2	26.7 (4.2)	25.4 (4.1)	27.5 (4.0)	27.1 (4.9)	29.9 (6.5)	30.0 (8.1)
Total Cholesterol, mmol/L	5.19 (0.95)	4.93 (0.86)	5.51 (0.92)	5.67 (0.92)	5.09 (1.13)	4.90 (0.96)
Triglycerides mmol/L	1.65 (1.13)	1.19 (0.66)	1.47 (0.84)	1.26 (0.64)	1.82 (1.43)	1.29.(076)
HDL-Cholesterol, mmol/L	1.21 (0.28)	1.44 (0.33)	1.43 (0.38)	1.71 (0.43)	1.13 (0.31)	1.34 (0.33)
LDL-Cholesterol, mmol/L	3.28 (0.82)	2.95 (0.73)	3.47 (0.88)	3.39 (0.85)	3.37 (1.00)	3.15 (0.87)
Systolic BP, mmHg	136.2 (13.1)	124.3 (13.9)	141.1 (20.5)	136.1 (22.4)	121.2 (14.0)	114.2 (14.6)
Diastolic BP, mmHg	84.8 (10.8)	80.2 (10.5)	87.3 (10.7)	84.0 (9.6)	81.7 (10.1)	77.0 (10.0)
CIMT, mm	0.644	0.613	0.830	0.788	0.810	0.741
	(0.106)	(0.086)	(0.193)	(0.167)	(0.184)	(0.156)
CAE, diameter change	1.740	2.020	0.880	0.946	-	-
(%)/10 mmHg	(0.614)	(0.729)	(0.417)	(0.522)		

All units are expressed as mean (SD) except for dichotomous variables.

Abbreviations: CIMT, Carotid artery Intima Media Thickness; CAE Carotid Artery Elasticity; HDL, High Density Lipoprotein; LDL, Low Density Lipoprotein; BP, Blood Pressure; BMI, body mass index.

### The association between GRS_24SNP/CAD_ and subclinical atherosclerosis in the YFS

**Figure 1:**
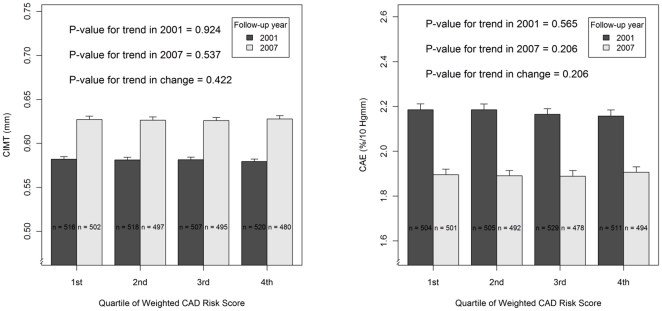
Subclinical atherosclerosis and the genetic predisposition to coronary artery disease (CAD). Carotid artery Intima Media Thickness (CIMT) and Carotid Artery Elasticity (CAE) among healthy adults with different genetic risk for coronary artery disease (CAD). The study population (The Cardiovascular risk in Young Finns Study) is stratified into four groups according to CAD risk allele dosage (calculated weighted risk score).

According to both unadjusted and adjusted linear regression analyses the GRS_24SNP/CAD_ did not associate with CIMT measured in 2007 and 2001 or with the 6-year incidence of high carotid atherosclerosis (Table 2 and Figure 1). The GRS_24SNP/CAD_ had no age-, sex- or BMI-dependent associations with CIMT (p = 0.327 for age-by-risk score interaction, p = 0.522 for sex-by-risk score interaction and p = 0.454 for BMI-by-risk score interaction). The progression of CIMT between 2001 and 2007 was also unaffected by the GRS_24SNP/CAD_ (Figure 1). Figure 1 demonstrates the mean CIMT values measured in 2001 and 2007 for each quartile when the study population was stratified according to risk allele dosage.

**Table 2:** The association between sub-clinical atherosclerosis measured from the carotid artery and the genetic risk score formed from 24 risk variants previously associated with coronary artery disease.

Phenotype	Beta*	S.E.	P-value
Intima Media Thickness			
(mm)			
Model 1	−0.000015	0.00069	0.982
Model 2	−0.00025	0.00063	0.694
Model 3a	−0.00036	0.00063	0.574
Elasticity			
(%/10 mmHg)			
Model 1	−0.00914	0.00496	0.065
Model 2	−0.00646	0.00459	0.160
Model3b	−0.0038	0.0045	0.397

	OR**	95% CI	P
6-year incidence of carotid			
atherosclerosis***			
Model 1	0.995	0.942 to 1.051	0.859
Model 2	1.000	0.945 to 1.058	0.997
Model 3a	0.998	0.941 to 1.057	0.936

Adjustments: Model 1 – unadjusted; Model 2 – age, sex and Body Mass Index; Model 3a – age, sex, body mass index, apoliprotein A, apolipoprotein B, systolic blood pressure and waist circumference; Model 3b – age, sex, body mass index, blood glucose, waist circumference and diastolic blood pressure.

*Beta for a change of one standard deviation in the risk score.

**For an increase of one risk allele in the GRS_24SNP/CAD_.

***The 6-year incidence of carotid atherosclerosis is defined as the occurrence of substantial CIMT (age-, sex- and BMI- adjusted CIMT- value over 90% percentile) or plaque in 2007 in subjects who did not have substantial CIMT or plaque in 2001.

Abbreviations: S.E., Standard Error; OR, Odds Ratio; CI, Confidence interval.

Similarly, the GRS_24SNP/CAD_ did not associate with carotid artery elasticity measured in 2007 although a borderline association was seen before adjusting the analysis with age, sex and BMI. Before any adjustments, a change of one standard deviation in the risk score seemed to associate with a lower elasticity (a −0.00914% change [S.E. of 0.00496] in the lumen diameter in response to a 10 mmHg increase in arterial blood pressure, p = 0.065). However, this observed difference diminished when age sex and BMI were added to the model (β = −0.00646%/10 mmHg with S.E. of 0.00459%/10 mmHg, p = 0.160). In comparison, in the same model, male sex corresponded to a 0.25014% decrease in elasticity in response to a 10 mmHg change in arterial blood pressure (S.E. of 0.02906, p = 1.5E^−17^). The GRS_24SNP/CAD_ was not observed to have any major age-, sex- or BMI-dependent effects on CAE (p = 0.151 for age-by-risk score interaction, p = 0.090 for sex-by-risk score interaction and p = 0.931 for BMI-by-risk score interaction). Correspondingly, the GRS_24SNP/CAD_ did not associate with CAE in 2001 or with the progression of CAE between 2001 and 2007 (Figure 1).

When analysed separately, the individual SNPs did not have any dramatic effect on CIMT or CAE measured in 2007. The associations by all 24 genotypes are summarized in Table S1 and Table S2. Only one SNP (rs4977574) was seen to associate significantly with CIMT (p = 0.010) and only one variant (rs4773144) associated significantly with CAE (p = 0.008). These associations were not successfully replicated in any of the replication cohorts (Tables S3, S4, and S5).

### The performance of CAD SNPs in the prediction of extreme CIMT in the YFS

Without any adjustments, the predictive value of the 24 SNPs was not high (area under the receiver operating characteristic curve [AUC] 0.604). Age, sex and BMI alone predicted extreme CIMT better (AUC 0.831). Adding the few CAD SNPs with any predictive effect on CIMT to the model did not seem to the increase the prediction accuracy (AUC 0.838).

We have also showed previously that the group of SNPs with the highest predictive value for extreme CIMT in the YFS population in 2001 predicts extreme CIMT with an even greater accuracy in 2007 [23]. For this reason, we also whether there would be any consistency in the results obtained in 2001 and 2007. According to a machine learning –based SNP selection adjusted with age, sex and BMI, the same CAD SNPs that were observed to provide some predictive value for extreme CIMT in 2001 did not seem to predict extreme CIMT in 2007 (Table S6).

### Replication and the meta-analysis

In the Bogalusa Heart Study, genotype information was available for 23 variants from a population of European ancestry and for 19 variants from a population of African ancestry. The genetic risk score was formed accordingly from the available variants for both populations separately. In line with the results of the YFS, the GRSs thus formed did not associate with CIMT in the populations of European or African ancestry. The analyses were repeated with no adjustments and by adjusting for age, sex and BMI, in addition to adjusting for serum lipid values (total cholesterol, LDL cholesterol, HDL -cholesterol and triglycerides) and as well as systolic and diastolic blood pressure, accepting only significant covariates into the final model by a stepwise procedure (Table 3).

**Table 3:** The association between carotid intima media thickness and genetic risk score (GRS) among two different study populations of the Bogalusa Heart Study.

	BHS European Ancestry (n = 755)			BHS African Ancestry (n = 326)		
	Beta*	S.E.	P-value	Beta*	S.E.	P-value
GRS						
Model 1	−0.00054	0.00198	0.785	0.00252	0.00455	0.580
Model 2	−0.00246	0.00180	0.172	0.00344	0.00427	0.421
Model 3a/b	−0.00277	0.00174	0.111	0.00346	0.00420	0.420

The GRS was calculated as a weighted sum of coronary artery disease risk alleles from 24 known variants.

Adjustments: Model 1 –unadjusted; Model 2 – age, sex and Body Mass Index; Model 3a (European ancestry) – age, sex, body mass index, total cholesterol, low density lipoprotein -cholesterol, triglycerides and systolic blood pressure; Model 3b (African ancestry) – age, sex, body mass index, and systolic blood pressure. Abbreviations: GRS, Genetic Risk Score, S.E., Standard Error.

*Change of CIMT in millimetres corresponding to a change of one standard deviation in the risk score.

Only few of the SNPs associated individually with CIMT in the separate replication cohorts and none associated in the meta-analyses (Tables S3, S4, S5 and S7). None of the five genotyped SNPs in the Health 2000 Survey associated with CAE (Table S3).

## Discussion

We found that a genetic risk score formed of variants previously associated with the risk of CAD on a genome-wide -significant level (SNPs) does not predict the development of subclinical atherosclerosis measured as intimal thickening, the arterial elasticity of the carotid artery, or the incidence of high CIMT or plaques during a six-year follow-up among healthy adults. The results of the present study also showed that individual polymorphisms do not have a substantial impact on the development of subclinical atherosclerosis. The few observed significant associations were moderate at best and were not replicable. A meta-analysis of three independent study populations confirmed the negative results.

CIMT is a good non-invasive method for evaluating the development of atherosclerosis and it is associated with the risk of CAD [24], [25], [26]. In apparently healthy young individuals, it provides an excellent means to follow the development of the early phases of atherosclerosis. A recent meta-analysis revealed that an approximately 0.1 mm change in CIMT corresponds to an increase of 15% in the risk of myocardial infarction [17]. In the present study, the main cohort (YFS) alone had an 80% power to detect at least 0.0155 mm changes for all SNPs in assuming additive genotypic effects, which should therefore be enough to reveal an at least 2.3% increase in the risk of MI. In the present study, the genetic risk score did not associate with increased CIMT. Previously, each of the studied 24 SNPs have been associated with a 6%–29% increase in the risk of CAD per one risk allele, depending on the variant [12]. In the YFS population, the maximal significant difference in individual genotypic means was 0.013 mm, and it was not replicated in other cohorts.

Although similar GRSs have shown potential for being an effective tool in risk stratification for CAD [27], [28], [29], the CAD GRS used in the present study does not seem to have any association with CIMT or CAE measured in a cross-sectional study setting or with the development of these end-points in a six-year follow-up. In the case of the GRS in the YFS, we achieved an 80% power to detect an at least 0.017 mm difference in CIMT and a 0.120%/10 mmHg difference in CAE between the individual population groups when the YFS population was divided into four categories/quartiles by CAD risk score dosage. The 0.017 mm difference in CIMT would correspond to a maximum of 2.6% increase in the risk for MI (as discussed above). As for CAE, the power of the current study was sufficient to detect a difference of less than half (0.120%/10 mmHg) of the observed difference between men and women in CAE (0.280%/10 mmHg) when comparing any two quartiles. Considering our findings (or the lack of significant associations), it is plausible to conclude that the studied polymorphisms would have some other more powerful ways of mediating the risk for CAD than through early intimal thickening and decreased arterial elasticity, at least when measured from the carotid artery among healthy adults. However, the results of GRSs should be interpreted with caution especially when only 24 variants are used to form a risk score. Thus far, genetic risk scoring for Crohn's disease, Bipolar disorder and even CAD, has been shown effective in risk prediction, provided that a sufficiently large amount (hundreds or even thousands) of variants is used (even if their individual effects would be merely nominal) [27], [30], [31]. We have previously shown in the YFS population that it is possible to predict, with good accuracy, the risk for extreme CIMT by using multiple SNPs of good predictive value even though they would not associate statistically significantly with CIMT. In fact, when combined with traditional risk factors, the SNPs with high predictive value in a multivariate model (although not statistically significantly associating with CIMT) predicted the risk of extreme CIMT better (AUC of 0.844) than traditional risk factors alone (AUC of 0.741). This method of comparing low and high extremes of quantitative traits has been shown to provide good statistical power for the studied variants [32], [33], [34]. In the present study, the CAD SNPs did not seem to increase the predictive value of the analysis when added to the model with age, sex and BMI Supporting our conclusion, these CAD variants have not been associated with CIMT or higher risk of carotid plaque in a recent GWAS published during the preparation of the current manuscript [35]. However, the application of the results of genetic association studies and especially the use of genetic risk scores has proved difficult in regard to most diseases with a complex etiology [31]. Confounding factors such as different phenotypic diversity within one disease group, as is the case with coronary heart disease, gene–gene interactions and gene–environment interactions as well as the modulating effect of time on genetic risk factors are challenging.

Thus far only few studies have investigated the role of individual SNPs associated with CAD in previous GWASs. The study by Cunnington et al. failed to find any association between CIMT and three individual CAD variants (rs1333049, rs6922269 and rs2943634) [19]. These results are in line with the meta-analysis results of the present study. We have also published a negative result between the SNP rs1333049 and CIMT based on the earlier measurements of the present YFS population from 2001 (at age 23–39 years) [21]. However, it is possible that some risk variants with modest effect can only be discovered in older populations with a heavier risk factor burden and more advanced CIMT changes. This could explain the positive association (if not due to chance) between CIMT and rs4977574 in 2007. The rs4977574 is in high linkage disequilibrium with rs1333049 which in 2001 was not observed to associate with CIMT. Supporting this, Ye et al. have found rs1333049 to associate with CIMT among substantially older subjects with a mean age of over 50- years. They also found a positive connection between this variant and the progression of CIMT during a 10- year follow-up [22].

At the time of the review process for the present publication, another study was published with results supporting our findings. The study by Conde et al. presented a meta-analysis of the association between CIMT and eleven independent variants previously associated with CAD in GWASs [36]. However, as the meta-analysis also included the YFS population, the results of the study by Conde et al. cannot be considered completely independent of our current results. Although eight of the variants overlap with the SNPs in the present publication, the SNP selection of the present study is more extensive (based on the results of the recent and thus far largest GWAS by the CARDIoGRAM consortium). Furthermore, the current study uniquely presents data from several measurements over time, thus also enabling the investigation of the possible association between individual variants associated with CAD on a genome-wide significant level and the development of CIMT and CAE. No other study has been published concerning the possible connection between the development of subclinical atherosclerosis and an extensive genetic risk score for CAD formed by using a similar selection of SNPs.

In 2007 the present study population consisted of six age groups between the ages of 30 and 45 years. As it is possible that with increasing age the effect of hereditary risk factors could change, we also screened for possible age-by-CAD risk score interactions affecting CIMT and CAE. Although, the interaction analyses showed no apparent age-dependent associations in the YFS or BHS, the possibility that age would modulate the effect of the individual genetic factors in older age groups or among populations with a heavier atherosclerotic burden cannot be excluded. A good example of this is the SNP rs501120 (locus 10q11.2) which is located upstream of the CXCL12 gene and is in complete linkage disequilibrium with rs1746048 identified as a risk variant for CAD by the CARDIoGRAM consortium [12]. In the current study population of young adults, rs1746048 did not associate significantly with CIMT or CAE. However,rs501120 and rs1746048 did seem to associate with CIMT among the African American population of the Bogalusa Heart Study as well as among the study population of the Health 2000 Survey where only rs501120 was genotyped. Both of these study populations have higher mean CIMT values when compared to the study population of the YFS orthe subjects of European ancestry in the BHS. The possible confounding effect of genetic heterogeneity can also explain some of the replication failures between these samples. Furthermore, we have also shown in a larger meta-analysis integrating three independent cohorts with a mean age over 50- years (Bruneck, Health2000 and HTO), that rs501120 associates with CIMT. These separate cohorts are based on older populations (Bruneck with an age range of 45–94 years, Health2000 with an age range 46–76 years, and HTO with and age range 19–88 years) [20].

The foremost problem of the present study is that we lacked the replication data for some of the SNPs in the replication cohorts, especially in the Health 2000 Survey. Therefore, we were unable to evaluate the effect of the risk score on carotid artery elasticity. However, we were able to replicate the findings regarding CIMT for most of the variants, and the consistency of the results obtained from each study population makes the interpretation of the main results relatively safe. The main strength of the study is the size of the combined study population (4,581 subjects with measurements of CIMT and 3,401 with measurements of CAE) and the extensiveness of the risk factor data in each cohort. Furthermore, the YFS population comprises randomly selected healthy individuals from the general population. Therefore, the study is free of selection bias. The YFS also provides a perfect opportunity to follow the development of subclinical atherosclerosis among healthy young adults because both CAE and CIMT have been measured from the same population twice six years apart (2001 and 2007). Although the Health 2000 survey population also represents a randomly selected general population, it is not entirely free of selection bias as the mean age is older (∼58 years) and cardiovascular mortality is likely to affect the study population. Fortunately, we were able to replicate our findings in the BHS with measurements of healthy adults of the same age as in the YFS. Due to the fact that the study populations of the YFS and BHS are relatively young (mean age ∼38 years in both), the result cannot be applied to older populations. Although the interaction analyses did not suggest that age would clearly modulate the effect of the genetic risk score on sub-clinical carotid atherosclerosis, it is possible that, in older populations, the association would be significant, as could also be the case for individual SNPs. We withheld from further age or sex stratifications in the current study population due to the negative results of the interaction analyses. Furthermore, such stratifications would have greatly decreased the power of the study. We also did not analyse the possible interactions between individual SNPs and risk factors in order to limit the number of analyses.

While the 24-SNP risk score did not identify subjects with increased subclinical risk for atherosclerosis, it is still quite possible that factors associated with pre-symptomatic stages of the disease could be used for identifying patients more predisposed to severe endpoints. Thus, in the future more precise methods are required for studying the mechanism (gene-by-gene interactions and gene-by-environment interactions) by which these risk factors influence the development of the disease. Furthermore, although CIMT and CAE are excellent surrogate phenotypes for studying the development of subclinical atherosclerosis (the early stages of intimal thickening), their results should be interpreted with caution as not all etiological factors are identical in the pathogenesis of coronary atherosclerosis. Many of the genetic risk factors might have different effects in different vascular beds and the phenotypes they promote are diverse as well (from stable manifestations of coronary artery disease to the most severe forms of acute coronary syndromes).

In conclusion, most of the studied SNPs should not influence CAD risk through a mechanism that would also affect the development of subclinical atherosclerosis measured by CAE or CIMT in a healthy general population of young adults.

## Materials and Methods

### Ethics statement

The study was approved by the institutional review board and the ethics committee of each participating institution (The Universities of Helsinki, Turku, Tampere, Oulu and Kuopio and the Institutional Review Board of the Tulane University Health Sciences Center). Written informed consent was obtained from each participant in accordance with institutional requirements and the principles expressed in the Declaration of Helsinki. All subjects gave informed consent at each examination, and for those under 18 years of age, the consent of a parent/guardian was obtained.

### The Cardiovascular Risk in Young Finns Study (YFS)

The YFS is an ongoing multicentre study with a randomly selected population of Caucasian adults. The study was launched in 1980 to assess risk factors underlying cardiovascular disease in children and young adults. The first cross-sectional study was conducted in 1980 and included 3,596 children and adolescents aged 3 to 18 years (six equal age cohorts: 3, 6, 9, 12, 15 and 18 years). Five university hospitals in Finland (Turku, Tampere, Helsinki, Kuopio, and Oulu) are participating in the study. The risk factor data was collected and CIMT and CAE measured in the follow-up studies in 2001 (n = 2265) and 2007 (n = 2197). All subjects gave written informed consent, and the study was approved by the local ethics committee and the study was conducted according to the principles expressed in the Declaration of Helsinki. The details of this study have been described earlier more extensively [14].

### Clinical Measurements and Questionnaires

Body height, weight, and waist circumference were measured and body mass index (BMI) was calculated. Blood pressure was measured thrice with the aid of an automated sphygmomanometer and the average of the measurements was used in the analyses. Subjects were also asked to complete questionnaires that included questions about smoking habits. Those smoking daily were considered current smokers. Biochemical measurements were performed following standard protocols (Data S1).

### Measurement of subclinical atherosclerosis

In both 2001 and 2007, the ultrasound studies were performed by trained sonographers following a standardized protocol (high resolution ultrasound imaging system [Sequoia 512, Acuson]). The CIMT and CAE measurements were later derived from the recorded scans by one experienced reader blinded to the subjects' clinical characteristics. Maximal and mean CIMT of the left carotid artery (the mean of 4 measurements 10 mm proximal to the carotid bifurcation) were recorded. CAE values were determined by selecting the best-quality cardiac cycle from the clip images and calculating the mean relative diameter change in carotid diameter (from end-diastole and end-systole) in response to a concomitant brachial artery blood pressure change. CAE = [(Ds – Dd)/Dd]/(Ps – Pd), where Dd is diastolic diameter, Ds is systolic diameter, Ps is systolic blood pressure, and Pd is diastolic blood pressure. The carotid diameter was measured at least twice (spatial measurements). The mean of the measurements was used as the end-diastolic or end-systolic diameter. Concomitant brachial blood pressure was measured with an automated sphygmomanometer (Omron M4, Omron Matsusaka Co., Ltd, Japan). To assess intra-individual reproducibility of ultrasound measurements, 57 subjects were re-examined three months after the initial visit in 2001 (2.5% random sample). The between-visit coefficient variation was 6.4% for CIMT and 16.3% for CAE.

### Genotyping and quality control of analyses

Genome-wide SNP data was obtained from 2,442 participants (1,123 males, 1,319 females) with a custom-built Illumina BeadChip Human670K including 546,677 SNPs. Genotypes were called using the Illuminus clustering algorithm [37]. Details of all experimental methods are described in Data S1.

### Replication Cohorts

The Bogalusa Heart Study (BHS): Between 1973 and 2008, 9 cross-sectional surveys of children aged 4–17 years and 10 cross-sectional surveys of adults aged 18–48 years who had been previously examined as children were conducted for CVD risk factor examinations in Bogalusa, Louisiana. This panel design of repeated cross-sectional examinations has resulted in serial observations from childhood to adulthood. By linking the 19 surveys, 12,163 individuals have been examined, with 37,317 observations. In the ongoing Longitudinal Aging Study funded by the NIH and NIA since 2000, 1,202 subjectshave been examined 4–14 times from childhood to adulthood and have DNA available for GWA genotyping. The genotyping methods and ultrasound study protocols have been described earlier in detail [38], [39].

Health 2000 cohort: The Health 2000 Survey was a large Finnish cross-sectional health examination survey carried out in 2000–2001. The overall study cohort was a 2-stage stratified cluster sample (8,028 persons) representing the entire Finnish population aged 30 years and above (Aromaa A, Koskinen S, Eds. Health and functional capacity in Finland. Baseline results of the Health 2000 Health Examination Survey. Helsinki: Publications of the National Public Health Institute B12/2004; 2004. Available at: www.ktl.fi/health2000). To study cardiovascular disease risk factors and diabetes more thoroughly, a supplemental study was carried out (sample size 1867 and participation rate 82%). The subjects in the supplemental study were aged 45 years and older, and the study was conducted in the catchment areas of the five Finnish University Hospitals. Carotid ultrasound examination was included in the supplemental study. There were 1,291 subjects (572 men and 719 women; mean age 58 years; range, 46–76 years) with available carotid ultrasound data. In the present study, we used measurements of the mean CIMT and CAE of the common carotid artery. In the Health 2000 survey, genomic DNA was extracted from peripheral blood leukocytes and genotyping was performed by using Taqman SNP Genotyping Assays and the ABI Prism 7900HT Sequence Detection System (Applied Biosystems, Foster City, CA, USA). The study protocol was approved by the Ethics Committee of the National Public Health Institute and carried out according to the recommendations of the Declaration of Helsinki. The details of the Health 2000 survey are described in more detail in Data S1.

### Formation of the genetic (CAD) risk score

We calculated a multi-SNP risk score (GRS_24SNP/CAD)_ summarizing the impact of 24 independent CAD- and MIrelated SNPs for CIMT and CAE. The GRS_42SNP/CAD_ was calculated as follows: for the i-th SNP in the j-th individual denote x_ij_ as the 0/1/2 coded genotype (for directly genotyped markers) or expected allele count (real values ranging between 0.0 and 2.0 for imputed markers). The risk score for participant j is then defined to be: s_j_ = w_1_x_1j_+w_2_x_2j_+…+w_m_x_mj_, where coefficients w_1_, w_2_,…w_m_ are specified to be the effect sizes, i.e., ORs, for CAD and MI from reported GWAS [26]. Besides using the weighted risk score, we also repeated the analyses using a crude risk score without weighing the allelic effects and including all SNPs in the score. This procedure did not alter the results in any significant manner and therefore only the effects of the weighted risk score are reported. In the BHS cohorts, genotypic information was available for 23 SNPs in the population of European ancestry and for 19 SNPs in the population of African ancestry. Similar GRSs were calculated separately for both populations using the genotypic information of these 23 and 19 SNPs.

### Statistical analysis

The endpoints of the YFS were CIMT, CAE, 6-year incidence of carotid atherosclerosis as well as CIMT and CAE progression during the follow-up. The 6-year incidence of carotid atherosclerosis was defined as the occurrence of substantial CIMT (age-, sex- and BMI-adjusted CIMT -value over 90% percentile) or plaque in 2007 in subjects who did not have substantial CIMT or plaque in 2001. Due to the normal distribution of the CIMT and CAE data in the present study, linear regression was used to investigate the possible connection between GRS_24SNP/CAD_ and subclinical atherosclerosis. As it was not clear whether CAD risk score would have a linear effect on the end-points, the analyses were also repeated after stratification of study population into four equal groups (quartiles). All analyses were first performed without adjustments (Model 1) and secondly adjusting for age, sex and BMI (Model 2). In the last phase the analyses were adjusted for all of the risk factors significantly associating with CIMT in a multivariable regression analysis (Model 3). Covariates were selected/filtered by a stepwise procedure. In the YFS the following covariates were finally used for CIMT: age, waist circumference, systolic blood pressure, Apolipoprotein A1 and ApolipoproteinB. For CAE we used systolic blood pressure, age, waist circumference, diastolic blood pressure, BMI and glucose. A similar protocol was also applied when screening for possible associations between GRSs and CIMT among the study populations of the BHS. Finally, in the YFS linear regression model adjusted with age, sex and BMI was employed when screening for as age-group-by-risk score interaction (study population divided into two equal groups), a sex-by-risk score interaction and a BMI-by-risk score interaction.

In all study cohorts, analysis of variance (ANOVA) adjusted with age, sex and BMI was used to screen for associations between individual SNPs and the endpoints. The associations were also screened by exploring linear trends using linear regression analysis. Both methods were employed in order to increase the sensitivity of the screening. These analyses were not further adjusted because for most of the polymorphisms, the mechanisms by which they affect the risk of CAD are unknown. All homozygous carriers of any given minor genotype were additionally pooled with the heterozygotes, if the minor genotype represented less than 10% of the total population.

We have previously shown in the YFS population that it is possible to predict with good accuracy (using area under the receiver operating characteristic curve [AUC]), the risk for extreme CIMT by using multiple SNPs with good predictive value even though they would not associate statistically significantly with CIMT [23]. Therefore, a stratification to high and low risk groups was employed in order to increase the statistical power of the variants (e.g. to find positive associations with high sensitivity). This comparison means comparing two groups from both ends of the spectrum – with high and low CIMT values. We used the predefined and reported cut-off of 15% quintiles for CIMT. A more precise statistical is description available in the supplementary material.

Study power calculations were performed for the primary study population (YFS). Based on power analyses conducted for each SNP, we had at least 80% power at a P-value of 0.05 to detect a beta value of 0.01 mm for CIMT for most of the SNPs (15/24∼62.5%) and for all SNPs, we obtained a beta value of 0.0155 mm assuming additive genotypic effects. In recessive models, we had at least 80% power at a P-value of 0.05 to detect a beta value of 0.075 mm for CIMT for all SNPs. For CAE, we had at least 80% power at a P-value of 0.05 to detect a beta value of 0.074%/10 mmHg with most of the SNPs (15/24∼62.5%). For all of the SNPs, we have achieved 80% power at a P-value of 0.05 to detect a beta value of 0.112%/10 mmHg and 0.5370%/10 mmHg with an additive and a recessive model, respectively. In the YFS we had 80% power, on a two-sided 0.05 significance level, to detect an at least 0.017 mm difference in CIMT and a 0.120%/10 mmHg difference in CAE between individual population groups when the YFS population was divided into four categories/quartiles by CAD risk score dosage. Furthermore, we had an approximately 80% power to detect a change of 0.002 mm in CIMT and 0.014%/10 mmHg in CAE per one unit change in allele risk dosage. The statistical power calculation was performed using QUANTO version 1.2.4.

Finally, a meta-analysis integrating the results of the independent study populations (YFS, and BHS and, for some SNPs, also the Health 2000 survey) was performed by using linear regression analysis (assuming additive effects for genotypes).

In the YFS population, only 140 subjects were on antihypertensive and 43 on lipid lowering medication. However, as these factors could possibly confound the analyses, the analyses were repeated after the exclusion of these subjects. The results remained unchanged after the adjustments and therefore only the results before the exclusion are presented.

Most of the genotypes were extrapolated from imputed data and although the reliability of the imputed data was extremely high we also repeated the same analyses for the primary endpoints before pooling the genotypes from the imputed data. Previously each of the 24 reported SNPs has been associated with CAD at significance levels exceeding a stringent genome-wide threshold. In the present study, we considered a p-value of 0.05 statistically significant provided that the observed effect of the risk allele was in the same direction as in the original report. Further adjustment would have weakened the power of the study greatly. All analyses were performed using SPSS Statistics software (version 18.0; SPSS Inc.,Chicago, IL, USA) and the meta-analysis using the rmeta-package (version 2.16) in R software.

## Supporting Information

Table S1 **The associations between single nucleotide polymorphisms and subclinical atherosclerosis in the Young Finns Study population according to analysis of variance adjusted with age, sex and body mass index.** Abbreviations: MAF, Mean Minor allele frequency; CIMT, carotid intima-media thickness; CAC, carotid artery compliance. *Maximal difference observed between genotypes. **low imputation quality. ***Tagging rs17465637.
(DOCX)

Table S2 **The associations between single nucleotide polymorphisms and subclinical atherosclerosis in the Young Finns study according to analysis of variance adjusted with age, sex, body mass index and geographical components.** All homozygous carriers of any given minor genotype were pooled with the heterozygotes, if the minor genotype represented less than 10% of the total population. Abbreviations: MAF. Mean Minor allele frequency; CIMT, carotid intima-media thickness (mm); CAE, carotid artery elasticity (%/10 mmHg). *Maximal difference observed between genotypes.
(DOCX)

Table S3 **The associations between coronary artery disease single nucleotide polymorphisms and subclinical atherosclerosis measured as carotid artery intima media thickness (CIMT) and carotid artery elasticity (CAE) in the Health 2000 survey.** *Tagging rs1746048, **Taggin rs4977574, ***P-value calculated by ANOVA adjusted age, sex and body mass index.
(DOCX)

Table S4 **Analysis of variance of the associations between risk variants for coronary artery disease and carotid artery intima media thickness (CIMT) among the participants with European ancestry of the Bogalusa Heart Study.** Abbreviations: SNP, single nucleotide polymorphism; MAF, mean allele frequency; S.E. Standard error. *Low imputation quality **Tagging the rs17465637_C.
(DOCX)

Table S5 **Analysis of variance of the associations between risk variants for coronary artery disease and carotid artery intima media thickness (CIMT) among the participants of African ancestry of the Bogalusa Heart Study.** Abbreviations: SNP, single nucleotide polymorphism; MAF, mean allele frequency; S.E. Standard error. *Low imputation quality **Tagging the rs17465637_C.
(DOCX)

Table S6 **The predictive powers (AUC) of machine-learning-based predictive models for extreme CIMT and CAE classes in the study populations.** The evaluation of the AUCs is based on a naïve Bayesian classifier and 10-fold cross validation procedure. Predictive SNPs in a model are the most informative in terms of AUC measure identified by an attribute selection algorithm among the 24 CAD SNPs. Also the predictive power of models adjusted with only age sex and body mass index (risk factors, RFs) are presented.
(DOCX)

Table S7 **Meta-analysis of the effects of individual coronary artery disease variants using linear regression analysis adjusted with age, sex and body mass index.** *Tagging rs4977574 in the Health 2000 Survey**Tagging rs1746048 in the Health 2000 Survey***The percentage of the variation explained by heterogeneity Abbreviations: YFS, The Young Finns Study; BHS, Bogalusa Heart Study.
(DOCX)

Data S1 **Data supplement containing additional information of materials and methods used in the YFS and Health 2000 survey.**
(DOCX)
