# Supplementary material for: Genetic Profiling Using Genome-Wide Significant Coronary Artery Disease Risk Variants Does Not Improve the Prediction of Subclinical Atherosclerosis: The Cardiovascular Risk in Young Finns Study, the Bogalusa Heart Study and the Health 2000 Survey – A Meta-Analysis of Three Independent Studies
Source: PLoS One. 2012 Jan 25;7(1):e28931. doi: 10.1371/journal.pone.0028931 (PMC3266236; doi:10.1371/journal.pone.0028931)
Supplement: Table S1 — The associations between single nucleotide polymorphisms and subclinical atherosclerosis in the Young Finns Study population according to analysis of variance adjusted with age, sex and body mass index. Abbreviations: MAF, Mean Minor allele frequency; CIMT, carotid intima-media thickness; CAC, carotid artery compliance. *Maximal difference observed between genotypes. **low imputation quality. ***Tagging rs17465637. (DOCX) [file pone.0028931.s001.docx]

| Band | SNP  (coded allele) | Nearest gene(s) | MAF | Genotype (n) | Carotid IMT in millimeters (S.E.) | | | P |  | Carotid artery lumen diameter change in %/10mmHg (S.E.) | | |  |
| --- | --- | --- | --- | --- | --- | --- | --- | --- | --- | --- | --- | --- | --- |
|  |  |  |  |  | G1 | G2 | G3 |  |  | G1 | G2 | G3 | P |
| 1p32.3 | rs11206510 (T) | PCSK9 | 0.159 | 55/668/1718 | 0.633 (0.012) | 0.625 (0.004) | 0.627 (0.002) | 0.775 |  | 1.914 (0.088) | 1.870 (0.028) | 1.903 (0.017) | 0.586 |
| 19p13.2 | rs1122608 (G) | LDLR | 0.224 | 108/880/1454 | 0.630 (0.009) | 0.625 (0.003) | 0.628 (0.003) | 0.720 |  | 1.957 (0.067) | 1.889 (0.024) | 1.895 (0.019) | 0.638 |
| 7q32.2 | rs11556924 (C) | ZC3HC1 | 0.402 | 354/1254/834 | 0.634 (0.006) | 0.624 (0.003) | 0.628 (0.003) | 0.336 |  | 1.891 (0.047) | 1.929 (0.0.22) | 1.853 (0.025) | 0.143 |
| 6q23.2 | rs12190287** (C) | TCF21 | 0.446 | 442/1292/708 | 0.628 (0.005) | 0.625 (0.003) | 0.628 (0.004) | 0.353 |  | 1.911 (0.038) | 1.869 (0.022) | 1.875 (0.032) | 0.489 |
| 10q24.32 | rs12413409 (G) | CYP17A1, CNNM2, NT5C2 | 0.081 | 10/373/2059 | 0.647 (0.031) | 0.623 (0.005) | 0.627 (0.002) | 0.755 |  | 1.800 (0.244) | 1.854 (0.038) | 1.903 (0.015) | 0.440 |
| 6p24.1 | rs12526453 (C) | PHACTR1 | 0.316 | 252/1040/1150 | 0.640 (0.006) | 0.625 (0.003) | 0.627 (0.003) | 0.110 |  | 1.917 (0.046) | 1.902 (0.022) | 1.886 (0.021) | 0.496 |
| 17p11.2 | rs12936587 (G) | RASD1, SMCR3, PEMT | 0.339 | 266/1125/1051 | 0.622 (0.006) | 0.627 (0.003) | 0.627 (0.003) | 0.615 |  | 1.860 (0.044) | 1.891 (0.022) | 1.894 (0.022) | 0.914 |
| 1p32.2 | rs17114036 (A) | PPAP2B | 0.106 | 25/466/1951 | 0.627 (0.020) | 0.626 (0.004) | 0.627 (0.002) | 1.000 |  | 1.578 (0.145) | 1.922 (0.032) | 1.894 (0.016) | 0.113 |
| 10q11.21 | rs1746048 (C) | CXCL12 | 0.153 | 54/636/1747 | 0.628 (0.013) | 0.628 (0.004) | 0.624 (0.002) | 0.844 |  | 1.897 (0.099) | 1.929 (0.028) | 1.885 (0.017) | 0.406 |
| 6p21.31 | rs17609940 (G) | ANKS1A | 0.194 | 92/764/1586 | 0.620 (0.010) | 0.628 (0.004) | 0.627 (0.002) | 0.787 |  | 1.797 (0.071) | 1.883 (0.026) | 1.907 (0.018) | 0.282 |
| 17p13.3 | rs216172 (C) | SMG6, SRR | 0.346 | 1051/1090/301 | 0.628 (0.003) | 0.626 (0.003) | 0.626 (0.006) | 0.974 |  | 1.899 (0.021) | 1.906 (0.021) | 1.839 (0.042) | 0.474 |
| 14q32.2 | rs2895811 (G) | HHIPL1 | 0.427 | 805/1184/449 | 0.625 (0.003) | 0.630 (0.003) | 0.620 (0.005) | 0.159 |  | 1.916 (0.025) | 1.884 (0.021) | 1.884 (0.033) | 0.592 |
| 12q24.12 | rs3184504 (A) | SH2B3 | 0.400 | 897/1136/409 | 0.628 (0.003) | 0.628 (0.003) | 0.622(0.005) | 0.653 |  | 1.884 (0.024) | 1.903 (0.021) | 1.898 (0.035) | 0.827 |
| 15q25.1 | rs3825807 (A) | ADAMTS7 | 0.333 | 283/1061/1098 | 0.625 (0.006) | 0.629 (0.003) | 0.625 (0.003) | 0.624 |  | 1.863 (0.042) | 1.897 (0.022) | 1.900 (0.021) | 0.710 |
| 13q34 | rs4773144 (G) | COL4A1, COL4A2 | 0.399 | 858/1215/369 | 0.629 (0.004) | 0.629 (0.003) | 0.622 (0.005) | 0.233 |  | **1.945 (0.027)** | **1.859 (0.021)** | **1.901 (0.039)** | **0.008** |
| 9p21.3 | rs4977574 (G) | CDKN2A, CDKN2B | 0.433 | 772/1221/447 | **0.620 (0.004)** | **0.632 (0.003)** | **0.624 (0.005)** | **0.010** |  | 1.935 (0.026) | 1.882 (0.020) | 1.862 (0.033) | 0.158 |
| 9q34.2 | rs579459 (C) | ABO | 0.230 | 1443/875/124 | 0.626 (0.003) | 0.630 (0.003) | 0.620 (0.009) | 0.412 |  | 1.891 (0.019) | 1.900 (0.024) | 1.907 (0.063) | 0.940 |
| 1p13.3 | rs599839 (A) | SORT1 | 0.217 | 122/817/1503 | 0.613 (0.009) | 0.625 (0.003) | 0.629 (0.003) | 0.238 |  | 1.893 (0.064) | 1.908 (0.024) | 1.884 (0.018) | 0.811 |
| 2q33.1 | rs6725887 (G) | WDR12 | 0.118 | 1892/522/28 | 0.627 (0.002) | 0.627 (0.004) | 0.616 (0.018) | 0.838 |  | 1.901 (0.016) | 1.876 (0.031) | 1.896 (0.133) | 0.784 |
| 11q23.3 | rs964184 (C) | ZNF259, APOA5-A4-C3-A1 | 0.144 | 1796/588/58 | 0.628 (0.002) | 0.626 (0.004) | 0.608 (0.013) | 0.322 |  | 1.894 (0.017) | 1.890 (0.029) | 1.984 (0.095) | 0.632 |
| 21q22.11 | rs9982601 (T) | MRPS6 | 0.143 | 1796/594/52 | 0.626 (0.002) | 0.627 (0.004) | 0.637 (0.014) | 0.606 |  | 1.883 (0.017) | 1.943 (0.029) | 1.895 (0.100) | 0.188 |
| 17q21.32 | rs46522 (T) | UBE2Z, GIP, ATP5G1, SNF8 | 0.456 | 495/1235/711 | 0.627 (0.004) | 0.628 (0.003) | 0.625 (0.004) | 0.843 |  | 1.909 (0.032) | 1.876 (0.020) | 1.919 (0.027) | 0.383 |
| 3q22.3 | rs2306374 (C) | MRAS | 0.346 | 1049/1087/300 | 0.628 (0.002) | 0.623 (0.005) | 0.621 (0.020) | 0.668 |  | 1.898 (0.016) | 1.875 (0.034) | 2.016 (0.146) | 0.582 |
| 1q41 | rs17011666*** (A) | MIA3 | 0.188 | 76/736/1555 | 0.613 (0.011) | 0.628 (0.004) | 0.627 (0.002) | 0.532 |  | 1.962 (0.079) | 1.923 (0.026) | 1.883 (0.018) | 0.327 |
|  | | | | | | | | | | | | | |

|  |
| --- |
