# Supplementary material for: Genetic Profiling Using Genome-Wide Significant Coronary Artery Disease Risk Variants Does Not Improve the Prediction of Subclinical Atherosclerosis: The Cardiovascular Risk in Young Finns Study, the Bogalusa Heart Study and the Health 2000 Survey – A Meta-Analysis of Three Independent Studies
Source: PLoS One. 2012 Jan 25;7(1):e28931. doi: 10.1371/journal.pone.0028931 (PMC3266236; doi:10.1371/journal.pone.0028931)
Supplement: Table S2 — The associations between single nucleotide polymorphisms and subclinical atherosclerosis in the Young Finns study according to analysis of variance adjusted with age, sex, body mass index and geographical components. All homozygous carriers of any given minor genotype were pooled with the heterozygotes, if the minor genotype represented less than 10% of the total population. Abbreviations: MAF. Mean Minor allele frequency; CIMT, carotid intima-media thickness (mm); CAE, carotid artery elasticity (%/10 mmHg). *Maximal difference observed between genotypes. (DOCX) [file pone.0028931.s002.docx]

| SNP | Pooled (n) | CIMT  max difference* | P | CAE  max difference* | P |
| --- | --- | --- | --- | --- | --- |
| rs17114036 | 490/1952 | 0.000 | 0.997 | 0.016 | 0.659 |
| rs599839 | 938/1504 | 0.004 | 0.274 | 0.018 | 0.543 |
| rs17609940 | 856/1586 | 0.001 | 0.838 | 0.034 | 0.265 |
| rs579459 | 998/1444 | 0.003 | 0.478 | 0.010 | 0.731 |
| rs1122608 | 987/1455 | 0.003 | 0.498 | 0.001 | 0.973 |
| rs9982601 | 639/1803 | 0.002 | 0.639 | 0.056 | 0.082 |
| rs11206510 | 723/1718 | 0.002 | 0.656 | 0.028 | 0.378 |
| rs6725887 | 550/1892 | 0.001 | 0.899 | 0.024 | 0.486 |
| rs1746048 | 690/1747 | 0.002 | 0.570 | 0.042 | 0.190 |
| rs12413409 | 383/2059 | 0.003 | 0.616 | 0.051 | 0.207 |
| rs964184 | 646/1796 | 0.003 | 0.475 | 0.003 | 0.922 |
|  |  |  |  |  |  |
