# Supplementary material for: Genetic Profiling Using Genome-Wide Significant Coronary Artery Disease Risk Variants Does Not Improve the Prediction of Subclinical Atherosclerosis: The Cardiovascular Risk in Young Finns Study, the Bogalusa Heart Study and the Health 2000 Survey – A Meta-Analysis of Three Independent Studies
Source: PLoS One. 2012 Jan 25;7(1):e28931. doi: 10.1371/journal.pone.0028931 (PMC3266236; doi:10.1371/journal.pone.0028931)
Supplement: Table S3 — The associations between coronary artery disease single nucleotide polymorphisms and subclinical atherosclerosis measured as carotid artery intima media thickness (CIMT) and carotid artery elasticity (CAE) in the Health 2000 survey. *Tagging rs1746048, **Taggin rs4977574, ***P-value calculated by ANOVA adjusted age, sex and body mass index. (DOCX) [file pone.0028931.s003.docx]

| SNP | MAF | Genotype (n) | CIMT  Max difference (mm) | P*** |  | CAE  Max difference  (%/10mmHg) | P*** |
| --- | --- | --- | --- | --- | --- | --- | --- |
| rs599839 | 0.216 | 889/493/67 | 0.009 | 0.909 |  | 0.126 | 0.066 |
| pooled |  | 889/560 | 0.001 | 0.923 |  | 0.028 | 0.300 |
| rs501120* | 0.161 | 1032/386/42 | 0.042 | 0.127 |  | 0.041 | 0.530 |
| pooled |  | 1032/428 | 0.017 | 0.085 |  | 0.029 | 0.312 |
| rs4773144 | 0.424 | 505/674/282 | 0.017 | 0.209 |  | 0.009 | 0.949 |
| rs1333049** | 0.417 | 505/688/265 | 0.011 | 0.697 |  | 0.022 | 0.798 |
| rs10757278** | 0.417 | 507/692/264 | 0.005 | 0.914 |  | 0.032 | 0.675 |
