# Supplementary material for: Genetic Profiling Using Genome-Wide Significant Coronary Artery Disease Risk Variants Does Not Improve the Prediction of Subclinical Atherosclerosis: The Cardiovascular Risk in Young Finns Study, the Bogalusa Heart Study and the Health 2000 Survey – A Meta-Analysis of Three Independent Studies
Source: PLoS One. 2012 Jan 25;7(1):e28931. doi: 10.1371/journal.pone.0028931 (PMC3266236; doi:10.1371/journal.pone.0028931)
Supplement: Table S4 — Analysis of variance of the associations between risk variants for coronary artery disease and carotid artery intima media thickness (CIMT) among the participants with European ancestry of the Bogalusa Heart Study. Abbreviations: SNP, single nucleotide polymorphism; MAF, mean allele frequency; S.E. Standard error. *Low imputation quality **Tagging the rs17465637_C. (DOCX) [file pone.0028931.s004.docx]

| SNP | MAF | N | | |  | CIMT in mm (S.E.) | | |  |
| --- | --- | --- | --- | --- | --- | --- | --- | --- | --- |
|  |  | G1 | G2 | G3 |  | G1 | G2 | G3 | P |
| rs11206510_T | 0.199 | 35 | 231 | 489 |  | 0.753 (0.025) | 0.762 (0.010) | 0.757 (0.007) | 0.880 |
| rs1122608_G | 0.255 | 47 | 289 | 416 |  | 0.709 (0.021) | 0.758 (0.009) | 0.764 (0.007) | 0.052 |
| rs11556924_C | 0.401 | 120 | 365 | 270 |  | 0.755 (0.013) | 0.759 (0.008) | 0.758 (0.009) | 0.953 |
| rs12190287_C* | 0.372 | 81 | 332 | 251 |  | 0.770 (0.017) | 0.752 (0.008) | 0.766 (0.009) | 0.341 |
| rs12413409_G* | 0.089 | 5 | 105 | 645 |  | 0.742 (0.066) | 0.754 (0.014) | 0.759 (0.006) | 0.926 |
| rs12526453_C | 0.349 | 97 | 306 | 313 |  | 0.789 (0.014) | 0.755 (0.008) | 0.750 (0.008) | 0.075 |
| rs12936587_G | 0.481 | 164 | 356 | 191 |  | 0.741 (0.012) | 0.766 (0.008) | 0.762 (0.011) | 0.143 |
| rs17114036_A | 0.084 | 6 | 114 | 623 |  | 0.779 (0.060) | 0.758 (0.014) | 0.759 (0.006) | 0.939 |
| rs1746048_C | 0.133 | 13 | 175 | 566 |  | 0.757 (0.041) | 0.770 (0.011) | 0.755 (0.006) | 0.479 |
| rs17609940_G | 0.206 | 37 | 237 | 480 |  | 0.721 (0.024) | 0.767 (0.010) | 0.757 (0.007) | 0.208 |
| rs216172_C | 0.364 | 291 | 370 | 87 |  | 0.767 (0.009) | 0.748 (0.008) | 0.761 (0.016) | 0.356 |
| rs2895811_G | 0.419 | 261 | 354 | 138 |  | 0.765 (0.009) | 0.757 (0.008) | 0.747 (0.012) | 0.510 |
| rs3184504_A | 0.458 | 214 | 390 | 151 |  | 0.761 (0.010) | 0.0757 (0.007) | 0.759 (0.012) | 0.949 |
| rs3825807_A | 0.454 | 145 | 395 | 215 |  | 0.764 (0.012) | 0.758 (0.007) | 0.754 (0.010) | 0.805 |
| rs4773144_G | 0.462 | 215 | 383 | 157 |  | **0.778 (0.010)** | **0.744 (0.007)** | **0.766 (0.012)** | **0.016** |
| rs4977574_G | 0.493 | 197 | 347 | 208 |  | **0.771 (0.010)** | **0.744 (0.008)** | **0.771 (0.010)** | **0.041** |
| rs579459_C | 0.209 | 465 | 265 | 25 |  | 0.762 (0.007) | 0.749 (0.009) | 0.790 (0.029) | 0.279 |
| rs599839_A | 0.237 | 43 | 272 | 439 |  | 0.730 (0.022) | 0.764 (0.009) | 0.757 (0.007) | 0.288 |
| rs6725887_G | 0.114 | 600 | 138 | 17 |  | 0.760 (0.006) | 0.757 (0.012) | 0.710 (0.036) | 0.383 |
| rs964184_C | 0.128 | 573 | 171 | 11 |  | 0.760 (0.006) | 0.758 (0.011) | 0.664 (0.044) | 0.099 |
| rs9982601_T | 0.126 | 573 | 166 | 12 |  | 0.763 (0.006) | 0.744 (0.011) | 0.742 (0.042) | 0.293 |
| rs17011666_A | 0.180 | 22 | 222 | 496 |  | 0.732 (0.031) | 0.766 (0.010) | 0.751 (0.007) | 0.328 |
| rs2306374_C | 0.160 | 534 | 200 | 21 |  | 0.755 (0.006) | 0.769 (0.010) | 0.740 (0.032) | 0.417 |
|  |  |  |  |  |  |  |  |  |  |
