# Supplementary material for: Genetic Profiling Using Genome-Wide Significant Coronary Artery Disease Risk Variants Does Not Improve the Prediction of Subclinical Atherosclerosis: The Cardiovascular Risk in Young Finns Study, the Bogalusa Heart Study and the Health 2000 Survey – A Meta-Analysis of Three Independent Studies
Source: PLoS One. 2012 Jan 25;7(1):e28931. doi: 10.1371/journal.pone.0028931 (PMC3266236; doi:10.1371/journal.pone.0028931)
Supplement: Table S6 — The predictive powers (AUC) of machine-learning-based predictive models for extreme CIMT and CAE classes in the study populations. The evaluation of the AUCs is based on a naïve Bayesian classifier and 10-fold cross validation procedure. Predictive SNPs in a model are the most informative in terms of AUC measure identified by an attribute selection algorithm among the 24 CAD SNPs. Also the predictive power of models adjusted with only age sex and body mass index (risk factors, RFs) are presented. (DOCX) [file pone.0028931.s006.docx]

|  | CIMT 15% (<15% vs >85%) | | | | | CAE 15% (<15% vs. >85%) | | | | |
| --- | --- | --- | --- | --- | --- | --- | --- | --- | --- | --- |
|  | RFs | SNPs | RFs  + SNPs | Predictive SNPs | n | RFs | SNPs | RFs  + SNPs | Predictive SNPs | n |
| YFS 01 | 0.79 | 0.524 | 0.795 | rs3184504_A  rs12190287_C  rs579459_C  rs4773144_G | 455 | 0.828 | 0.519 | 0.836 | rs599839_A  rs12526453_C  rs4773144_G  rs3825807_A  rs216172_C  rs46522_T  rs9982601_T | 468 |
| YFS 07 | 0.831 | 0.604 | 0.838 | rs11206510_T  rs4977574_G  rs964184_C  rs2895811_G  rs12936587_G  rs9982601_T | 451 | 0.822 | 0.606 | 0.83 | rs4977574_G  rs1746048_C  rs12413409_G  rs964184_C  rs17114036_A  rs11556924_C  rs216172_C  rs9982601_T | 468 |
