# Supplementary material for: Genetic Profiling Using Genome-Wide Significant Coronary Artery Disease Risk Variants Does Not Improve the Prediction of Subclinical Atherosclerosis: The Cardiovascular Risk in Young Finns Study, the Bogalusa Heart Study and the Health 2000 Survey – A Meta-Analysis of Three Independent Studies
Source: PLoS One. 2012 Jan 25;7(1):e28931. doi: 10.1371/journal.pone.0028931 (PMC3266236; doi:10.1371/journal.pone.0028931)
Supplement: Table S7 — Meta-analysis of the effects of individual coronary artery disease variants using linear regression analysis adjusted with age, sex and body mass index. *Tagging rs4977574 in the Health 2000 Survey**Tagging rs1746048 in the Health 2000 Survey***The percentage of the variation explained by heterogeneity Abbreviations: YFS, The Young Finns Study; BHS, Bogalusa Heart Study. (DOCX) [file pone.0028931.s007.docx]

|  | **The YFS (n=2015)** | | |  | **The BHS European Ancestry (n=755)** | | |  | **The BHS African Ancestry (n=362)** | | |  | **The Health 2000 Survey (n=1291)** | | |  | **The Meta-analysis (n=4387-2770)** | | | | | | |
| --- | --- | --- | --- | --- | --- | --- | --- | --- | --- | --- | --- | --- | --- | --- | --- | --- | --- | --- | --- | --- | --- | --- | --- |
| **SNP** | **beta** | **S.E.** | **P** |  | **beta** | **S.E.** | **P** |  | **beta** | **S.E.** | **P** |  | **beta** | **S.E.** | **P** |  | **beta** | **S.E.** | **ci.lb** | **ci.ub** | **P** | **I^2***** |  |
| rs11206510_T | 0.000 | 0.004 | 0.938 |  | -0.002 | 0.009 | 0.800 |  | 0.016 | 0.021 | 0.455 |  | NA | NA | NA |  | 0.000 | 0.003 | -0.006 | 0.007 | 0.923 | 0.000 |  |
| rs599839_A | 0.005 | 0.003 | 0.125 |  | 0.004 | 0.009 | 0.671 |  | 0.003 | 0.017 | 0.859 |  | -0.002 | 0.008 | 0.823 |  | 0.004 | 0.003 | -0.002 | 0.010 | 0.159 | 0.000 |  |
| rs17011666_A | 0.001 | 0.004 | 0.846 |  | NA | NA | NA |  | NA | NA | NA |  | NA | NA | NA |  | 0.001 | 0.004 | -0.007 | 0.009 | 0.846 | NA |  |
| rs6725887_G | -0.001 | 0.004 | 0.789 |  | -0.011 | 0.011 | 0.342 |  | -0.026 | 0.038 | 0.506 |  | NA | NA | NA |  | -0.003 | 0.004 | -0.010 | 0.005 | 0.512 | 0.000 |  |
| rs2306374_G | -0.004 | 0.005 | 0.371 |  | 0.007 | 0.010 | 0.476 |  | NA | NA | NA |  | NA | NA | NA |  | -0.002 | 0.004 | -0.011 | 0.007 | 0.640 | 3.550 |  |
| rs12526453_C | -0.003 | 0.003 | 0.322 |  | -0.020 | 0.008 | **0.012** |  | 0.016 | 0.024 | 0.499 |  | NA | NA | NA |  | -0.008 | 0.007 | -0.022 | 0.007 | 0.309 | 57.599 |  |
| rs4977574_G | 0.003 | 0.003 | 0.223 |  | 0.001 | 0.007 | 0.913 |  | 0.013 | 0.018 | 0.447 |  | NA | NA | NA |  | 0.003 | 0.003 | -0.002 | 0.008 | 0.202 | 0.000 |  |
| rs1746048_C | -0.002 | 0.004 | 0.686 |  | -0.012 | 0.011 | 0.289 |  | 0.028 | 0.014 | **0.049** |  | NA | NA | NA |  | 0.003 | 0.010 | -0.017 | 0.022 | 0.798 | 68.557 |  |
| rs1333049_C** | 0.003 | 0.003 | 0.398 |  | -0.004 | 0.007 | 0.549 |  | 0.016 | 0.016 | 0.315 |  | 0.008 | 0.007 | 0.220 |  | 0.003 | 0.003 | -0.002 | 0.008 | 0.255 | 0.000 |  |
| rs10757278_G** | 0.003 | 0.003 | 0.387 |  | -0.005 | 0.007 | 0.538 |  | 0.009 | 0.017 | 0.582 |  | 0.008 | 0.007 | 0.233 |  | 0.003 | 0.003 | -0.002 | 0.008 | 0.289 | 0.000 |  |
| rs501120_T* | -0.002 | 0.004 | 0.674 |  | -0.013 | 0.011 | 0.241 |  | 0.034 | 0.015 | **0.019** |  | 0.018 | 0.009 | **0.039** |  | 0.008 | 0.009 | -0.011 | 0.026 | 0.410 | 77.759 |  |
| rs3184504_A | -0.002 | 0.003 | 0.380 |  | -0.001 | 0.008 | 0.891 |  | NA | NA | NA |  | NA | NA | NA |  | -0.002 | 0.003 | -0.007 | 0.003 | 0.382 | 0.000 |  |
| rs1122608_G | 0.001 | 0.003 | 0.784 |  | 0.017 | 0.009 | 0.057 |  | NA | NA | NA |  | NA | NA | NA |  | 0.007 | 0.008 | -0.008 | 0.022 | 0.375 | 64.534 |  |
| rs9982601_T | 0.003 | 0.004 | 0.461 |  | -0.020 | 0.012 | 0.091 |  | -0.024 | 0.023 | 0.294 |  | NA | NA | NA |  | -0.008 | 0.010 | -0.027 | 0.011 | 0.406 | 55.760 |  |
| rs17114036_A | 0.000 | 0.005 | 0.948 |  | 0.002 | 0.013 | 0.899 |  | 0.000 | 0.021 | 0.982 |  | NA | NA | NA |  | 0.000 | 0.004 | -0.008 | 0.008 | 0.987 | 0.000 |  |
| rs17609940_G | 0.000 | 0.003 | 0.910 |  | 0.003 | 0.009 | 0.729 |  | NA | NA | NA |  | NA | NA | NA |  | 0.001 | 0.003 | -0.006 | 0.007 | 0.819 | 0.000 |  |
| rs12190287_C | 0.001 | 0.003 | 0.875 |  | 0.002 | 0.009 | 0.859 |  | 0.007 | 0.033 | 0.841 |  | NA | NA | NA |  | 0.001 | 0.003 | -0.006 | 0.007 | 0.819 | 0.000 |  |
| rs11556924_C | -0.002 | 0.003 | 0.641 |  | 0.001 | 0.008 | 0.882 |  | 0.023 | 0.027 | 0.386 |  | NA | NA | NA |  | -0.001 | 0.003 | -0.007 | 0.005 | 0.786 | 0.000 |  |
| rs579459_C | 0.001 | 0.003 | 0.746 |  | -0.004 | 0.010 | 0.682 |  | 0.049 | 0.023 | **0.034** |  | NA | NA | NA |  | 0.002 | 0.003 | -0.005 | 0.008 | 0.658 | 3.152 |  |
| rs12413409_G | 0.003 | 0.005 | 0.601 |  | 0.005 | 0.014 | 0.708 |  | -0.005 | 0.026 | 0.844 |  | NA | NA | NA |  | 0.003 | 0.005 | -0.007 | 0.012 | 0.567 | 0.000 |  |
| rs964184_C | -0.004 | 0.004 | 0.261 |  | -0.013 | 0.011 | 0.266 |  | -0.005 | 0.016 | 0.733 |  | NA | NA | NA |  | -0.005 | 0.004 | -0.012 | 0.002 | 0.142 | 0.000 |  |
| rs4773144_G | -0.003 | 0.003 | 0.342 |  | -0.009 | 0.008 | 0.259 |  | -0.003 | 0.015 | 0.841 |  | 0.004 | 0.006 | 0.574 |  | -0.003 | 0.003 | -0.008 | 0.003 | 0.329 | 0.000 |  |
| rs2895811_G | -0.002 | 0.003 | 0.577 |  | -0.009 | 0.008 | 0.247 |  | 0.011 | 0.017 | 0.521 |  | NA | NA | NA |  | -0.002 | 0.003 | -0.007 | 0.003 | 0.414 | 0.000 |  |
| rs3825807_A | -0.001 | 0.003 | 0.751 |  | -0.005 | 0.008 | 0.544 |  | 0.003 | 0.022 | 0.884 |  | NA | NA | NA |  | -0.001 | 0.003 | -0.007 | 0.004 | 0.626 | 0.000 |  |
| rs216172_C | -0.001 | 0.003 | 0.675 |  | -0.007 | 0.008 | 0.381 |  | -0.032 | 0.016 | **0.049** |  | NA | NA | NA |  | -0.006 | 0.006 | -0.017 | 0.005 | 0.275 | 41.236 |  |
| rs12936587_G | 0.002 | 0.003 | 0.473 |  | 0.009 | 0.008 | 0.248 |  | -0.012 | 0.019 | 0.530 |  | NA | NA | NA |  | 0.003 | 0.003 | -0.003 | 0.008 | 0.328 | 0.000 |  |
| rs46522_T | -0.001 | 0.003 | 0.710 |  | NA | NA | NA |  | NA | NA | NA |  | NA | NA | NA |  | -0.001 | 0.003 | -0.007 | 0.004 | 0.710 | NA |  |
