# Supplementary material for: Genetic Profiling Using Genome-Wide Significant Coronary Artery Disease Risk Variants Does Not Improve the Prediction of Subclinical Atherosclerosis: The Cardiovascular Risk in Young Finns Study, the Bogalusa Heart Study and the Health 2000 Survey – A Meta-Analysis of Three Independent Studies
Source: PLoS One. 2012 Jan 25;7(1):e28931. doi: 10.1371/journal.pone.0028931 (PMC3266236; doi:10.1371/journal.pone.0028931)
Supplement: Data S1 — Data supplement containing additional information of materials and methods used in the YFS and Health 2000 survey. (DOCX) [file pone.0028931.s008.docx]

**DATA SUPPLEMENT**

Genetic profiling using genome-wide- significant coronary artery disease risk variants does not improve the prediction of subclinical atherosclerosis

The Cardiovascular Risk in Young Finns Study

*Authors: Hernesniemi et al.*

**METHODS**

**Biochemical measurements in the YF-study**

Venous blood samples were taken after the subject had fasted for 12 hours. Lipid determinations were done with the use of standard methods. Details of analytical procedures have been reported previously^1^. Low-density lipoprotein (LDL) cholesterol was calculated by the Friedewald formula for subjects with <4 mmol/L triglycerides^2^. Serum insulin concentrations were measured by microparticle enzyme immunoassay kit (Abbott Laboratories, Diagnostic Division, Dainabot). Glucose concentrations were analyzed enzymatically with a clinical chemistry analyzer (Olympus, AU400). Serum C-reactive protein (CRP) was analyzed by an automated analyzer (Olympus AU400) with a latex turbidimetric immunoassay kit (CRP-UL assay, Wako Chemicals, Neuss, Germany). The coefficient of variation or these measurements were: 2.2% for total cholesterol, 2.3% for high-density lipoprotein (HDL) cholesterol, 3.8% for serum triglycerides, CV 2.1% for insulin, 2.0% for glucose and 3.3% for CRP. The details of other biochemical measurements have been described earlier in more detail ^1^.

**GWAS in YF-study**

In the start of QC protocol there was 2556 samples in the intensity file, after initial clustering 2 subjects were removed (CR<0.90), thus the main clustering include 2554 subjects, from these 54 samples failed QC. Thus genotyping pipeline contained 2500 subjects. Addtionally, 54 were removed due to Sanger genotyping pipeline QC criteria (i.e., duplicated samples, heterozygosity, low call rate, or Sequenom fingerprint discrepancy). After genotyping pipeline QC the following filters were applied to the remaining data: MAF 0.01, GENO 0.05, MIND 0.05, and HWE 1e-6. 3 of 2500 individuals were removed for low genotyping (MIND > 0.05), 11766 markers were excluded based on HWE test ( p ≤ 1e-06), 7746 SNPs failed missingness test (GENO > 0.05 ), 34596 SNPs failed frequency test (MAF < 0.01 ) and one individual failed gender check. None were remove by subsequent heterozygosity check. New binary files were created after removing the individual which failed the sex-check and identity-by-descent (IBD) matrix was subsequently calculated in PLINK ^3^. There was 546770 SNPs and 2496 individuals at this point which were utilized to generate the genome file. There were 51 pairs of individuals with pi-hat greater than 0.2 thus these individuals removed due to possible relatedness. One of the pair was removed using greater missingness as criteria. After final frequency and genotyping running, there was 546677 SNPs available from sample of 2442 YF subjects. Genotype imputation was performed using MACH 1.0 and HapMap II CEU (release 22, NCBI build 36, dbSNP 126) samples as reference. After imputation there were 2,543,887 imputed SNPs available. SNPs with squared correlation between imputed and true genotypes ≥ 0.30 were considered well imputed.

**Clinical and biochemical characteristics in the Health 2000 survey**

Height and weight were measured and body mass index (BMI) calculated. Current smoking was evaluated with a questionnaire. Those who were currently smoking were defined as smokers and the rest of the subjects as non-smokers. Blood pressure was measured from the right arm. The measurement was taken three times with 1–2-minute intervals. The automatic digital Omron M4 oscillometric manometer (Omron Matsusaka Co, Japan, Omron Healthcare Europe B.V., Hoofddorp, the Netherlands) was used. The average of the three measurements was used in the analysis. HDL cholesterol, total cholesterol and triglyceride concentrations were determined enzymatically (Roche Diagnostics, GmbH, Mannheim, Germany for HDL; Olympus System Reagent, Hamburg, Germany for total cholesterol and triglycerides) with a clinical chemistry analyser (Olympus, AU400, Hamburg, Germany). High-sensitivity C-reactive protein (hs-CRP) concentrations were determined using a chemiluminescent immunometric assay (Immulite, Diagnostic Products Corporation, Los Angeles, CA, USA). LDL cholesterol was calculated with the Friedewald formula.

**Measurement of subclinical atherosclerosis in the Health 2000 survey**

Carotid ultrasound examination of the right carotid artery was performed according to a standardized protocol using a 7.5 MHz linear array transducer. The examinations were performed by centrally trained and certified sonographers at 5 study locations around Finland. CIMT measurements were performed off-line with the use of automated imaging processing software (PROWIN 23.1). One reader was responsible for reading all ultrasound images. Mean and maximum CIMT were calculated and the mean of the measurements was used in this study. The intra-reader reproducibility of the CIMT measurements was assessed by calculation of the CIMT twice from 571 randomly selected images of 108 study subjects several weeks apart. The coefficient of variation for CIMT was 9.2%. The mean difference of the 2 measurements was 0.001 mm (SD 0.123), and the intra-class correlation was 0.934 (P<0.001).

**Predictive modeling and assessment of AUCs for extreme CIMT and CAE**

Based on the concept of extreme selection strategy, the quantile points 15% and 85% of the CIMT distribution were used to define the low and high risk classes, respectively. The prediction whether a subject belongs to a high or low risk class was done on the basis of his or her SNP data whereas age, sex and body mass index were used as confounding risk factors. The selection of the predictors and the predictive modeling were based on a probabilistic prediction model, the so-called naïve Bayes classifier, because of its low computational cost and good performance in our previous study. The selection of genetic risk factors and the predictive modeling was performed with the Weka data mining platform (version 3.7.0; University of Waikato, New Zeeland). The selection of the risk factors was conducted with the aim of identifying a minimal set of predictive attributes for predicting the different risk classes. The attribute selection was performed to implement a wrapper-based feature selection. This used naïve Bayes classifier utilizing a backwards selection method and 5-fold cross-validation to select the optimal subset of variables by excluding those factors with least predictive power. The final prediction accuracy of the classifiers was assessed using the receiver operating characteristic (ROC) analyses. The overall accuracy of a classifier was summarized using the area under the ROC curve (AUC) measure evaluated using 10-fold cross-validation to avoid potential selection bias.

REFERENCES:

**1.** Juonala M, Viikari JS, Hutri-Kähönen N, Pietikäinen M, Jokinen E, Taittonen L, Marniemi J, Ronnemaa T, Raitakari OT. The 21-year follow-up of the Cardiovascular Risk in Young Finns Study: risk factor levels, secular trends and east-west difference. *J Intern Med.* 2004;255(4):457-468.

**2.** Friedewald WT, Levy RI, Fredrickson DS. Estimation of the concentration of low-density lipoprotein cholesterol in plasma, without use of the preparative ultracentrifuge. *Clin Chem.* 1972;18(6):499-502.

**3.** Purcell S, Neale B, Todd-Brown K, Thomas L, Ferreira MA, Bender D, Maller J, Sklar P, de Bakker PI, Daly MJ, Sham PC. PLINK: a tool set for whole-genome association and population-based linkage analyses. *Am J Hum Genet.* 2007;81(3):559-575.
